# IndelQuant: A Workflow for Cost-Efficient Amplicon Sequencing Analysis of Genome-Edited Chickens

**DOI:** 10.3390/ijms27209054

**Published:** 2026-10-13

**Authors:** Yujuan Duan, Ke Xu, Hao Zhou, Chuan He, Jinmei Ding, Chao Qin, Yan Zhang, Chengxiao Han, He Meng

**Affiliations:** 1 School of Agriculture and Biology, Shanghai Jiao Tong University, Shanghai 200240, China; 2 School of Chemistry and Chemical Engineering, Shanghai Jiao Tong University, Shanghai 200240, China; 3 Carilion Clinic, Roanoke, VA 24016, USA

**Keywords:** genome editing, chicken, CRISPR, amplicon sequencing, low-cost workflow

## Abstract

Accurate quantification of Clustered Regularly Interspaced Short Palindromic Repeats (CRISPR)/Cas9-induced insertions and deletions (indels) is essential in chicken genome-editing studies, where low editing efficiencies and mosaicism can complicate the assessment of target-site modifications. Existing assays often involve a trade-off between affordability and the sensitivity orsequence information needed for detailed analysis. Here, we introduce IndelQuant, a cost-efficient workflow integrating sample-specific barcode PCR, pooled amplicon library construction, AdapterRemoval3-based read processing, and CRISPResso2-based indel quantification. The workflow provides graphical and tabularsummaries of editing efficiency, indel positions, indel-size distributions, and sample-level mutation profiles. We evaluated the workflow in *TYR*-edited DF-1 cells and genome-edited chickens. In edited chickens, IndelQuant detected tissue-specific indel signals, most below 5%, while negative controls showed background frequencies from 0 to 0.03%. These findings indicate that IndelQuant retains the sequence-level information provided by amplicon sequencing while simplifying sample processing and analysis, offering a practical approach to routine screening for low-frequency and mosaic indels in poultry genome-editing studies.

## 1. Introduction

Chickens play important roles in agriculture, functional genomics, and bioreactor research, yet genome editing in this species remains technically challenging. In birds, the zygote is difficult to access within the oviduct, and a freshly laid egg contains a multicellular blastoderm of approximately 40,000–60,000 cells, precluding the pronuclear-injection approaches routinely used in rodents [1]. Consequently, avian genome editing has primarily relied on two strategies: (i) in vitro modification and transplantation of cultured primordial germ cells (PGCs), which requires specialized culture systems and involves competition between transplanted and endogenous PGCs for germline colonization [2], and (ii) direct in ovo delivery of viral or nonviral vectors, which often results in low-efficiency, mosaic editing [3,4]. Even when editing is successful, G0 animals are often chimeric, with editing frequencies varying considerably among tissues and individuals [5]. Although CRISPR/Cas9 has enabled precise genetic manipulation, low editing efficiency and mosaicism in G0 animals complicate accurate assessment of target-site modifications [1,2,3,6,7,8,9,10,11,12], underscoring the need for detection methods that can detect low-frequency, heterogeneous indels while preserving the underlying mutation sequences in poultry genome-editing experiments.

Methods for detecting and quantifying CRISPR-induced indels involve trade-offs between cost and analytical resolution. For example, T7 endonuclease I (T7E1) and Surveyor assays are inexpensive and rapid but have limited ability to quantify rare or mosaic indels [13,14,15,16]. Sanger sequencing-based approaches, such as TIDE and ICE, offer accessible editing estimates but are less-suitable for highly heterogeneous samples [15,17]. Amplicon next-generation sequencing (NGS) provides greater sensitivity and sequence resolution, but conventional workflows can require costly library preparation, complex command-line operations, and several separate tools for demultiplexing, editing quantification, and visualization [16,18,19,20,21,22]. Benchmarking studies have shown that T7E1 and Sanger-based assays often underestimate editing efficiency relative to amplicon sequencing [23,24,25], and that different indel-analysis tools can produce discrepant mutation profiles from the same dataset [26]. CRISPResso2 is widely used as a reference standard for amplicon-based quantification [22,27]. However, its routine use requires command-line operations and separate handling of demultiplexing and batch-management steps.

To address these challenges, we developed IndelQuant v0.1.1, a cost-efficient workflow for targeted indel quantification in genome-edited animals. IndelQuant integrates sample-specific barcode PCR, pooled amplicon library construction, demultiplexing with AdapterRemoval3, and indel quantification using CRISPResso2 [22,28]. The software provides graphical parameter, configuration, automated output monitoring, result parsing, visualization, and CSV export, reducing the need to assemble and manage separate command-line tools for routine screening. We first validated IndelQuant in *TYR*-edited DF-1 cells alongside a T7E1 assay and clone-based Sanger sequencing, and then applied it to *TYR*-edited chickens generated through adenoviral CRISPR-Cas9 delivery. This study focuses on a practical use case: detecting low-frequency and mosaic indels in edited chickens using a streamlined alternative to conventional amplicon NGS workflows, with the potential to reduce per-sample costs and analytical workload.

## 2. Results

### 2.1. Development of IndelQuant for Low-Cost Barcoded Amplicon Sequencing

**Figure 1:**
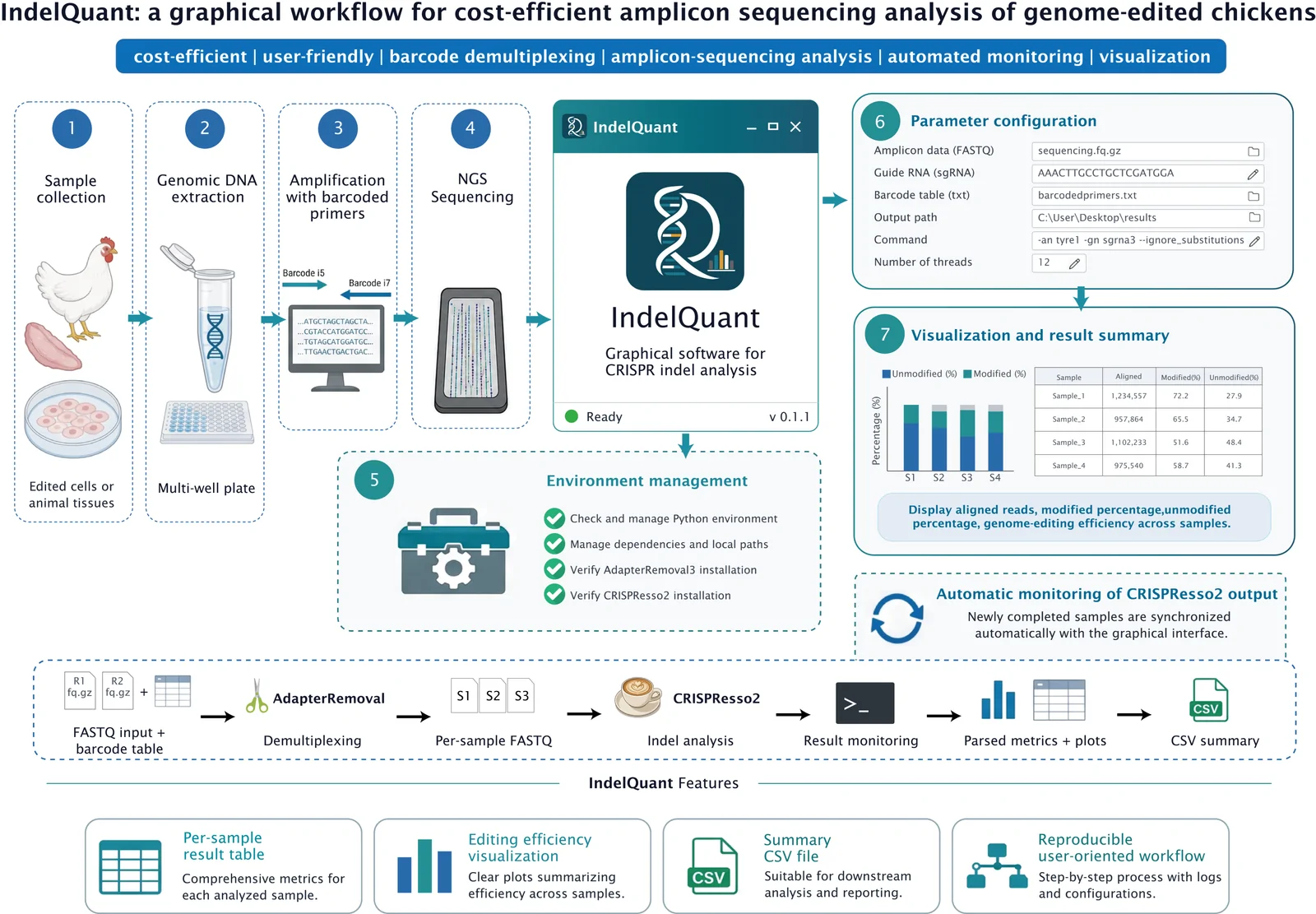
Overview of the IndelQuant workflow for low-cost amplicon sequencing analysis of genome-edited chickens. Genomic DNA is extracted from edited cells or chicken tissues and amplified using target-specific primers carrying sample-specific barcode pairs. Barcoded amplicons are pooled into a single sequencing library and subjected to paired-end amplicon sequencing. Raw reads are processed with AdapterRemoval3 for barcode-based demultiplexing, quality filtering, trimming, and paired-end read joining. The processed reads are subsequently analyzed with CRISPResso2 for reference-guided mapping and indel quantification. IndelQuant integrates these steps into a graphical workflow with environment checking, automated output monitoring, visualization, and CSV export.

To facilitate cost-efficient amplicon library construction, we used fusion primers containing unique 6-nt barcodes at the 5′ ends of both forward and reverse target-specific primers were used [29] (Figure 1). By combining six barcoded forward primers with six barcoded reverse primers, up to 36 uniquely labeled samples can be pooled for sequencing, facilitating sample identification while reducing the number of distinct barcode primers required. We used barcode sets recommended for the Illumina NovaSeq 6000 platform. Paired-end sequencing (PE250) was performed on amplicons of up to 450 bp to obtain sequence coverage around CRISPR cleavage sites.

IndelQuant was designed to streamline the complete analysis process (Supplementary Figures S1–S5). The software combines input-file selection, barcode-table configuration, AdapterRemoval3 execution, CRISPResso2 parameter setup, automated run monitoring, dependency and environment checks, result parsing, graphical visualization, and summary CSV export in a single user-friendly interface. Raw paired-end reads were first processed using AdapterRemoval3 [28], including barcode-based demultiplexing, removal of unassigned or unpaired reads, sequence trimming, and paired-end read alignment/merging. The processed reads were then analyzed using CRISPResso2 [22] for reference-guided mapping, indel quantification, and mutation-profile visualization.

During analysis, IndelQuant monitors CRISPResso2 output files and updates the graphical interface once valid results are available. It then summarizes editing efficiency, indel-position distribution, indel-size distribution, and sample-level mutation profiles. This integrated approach reduces the need to inspect multiple command-line output folders manually and simplifies post-sequencing analysis. Together, barcode-based pooling and graphical analysis provide a practical approach to low-frequency indel profiling, with reduced per-sample preparation costs and readily accessible read-level mutation information.

### 2.2. Validation of IndelQuant in TYR-Edited DF-1 Cells

**Figure 2:**
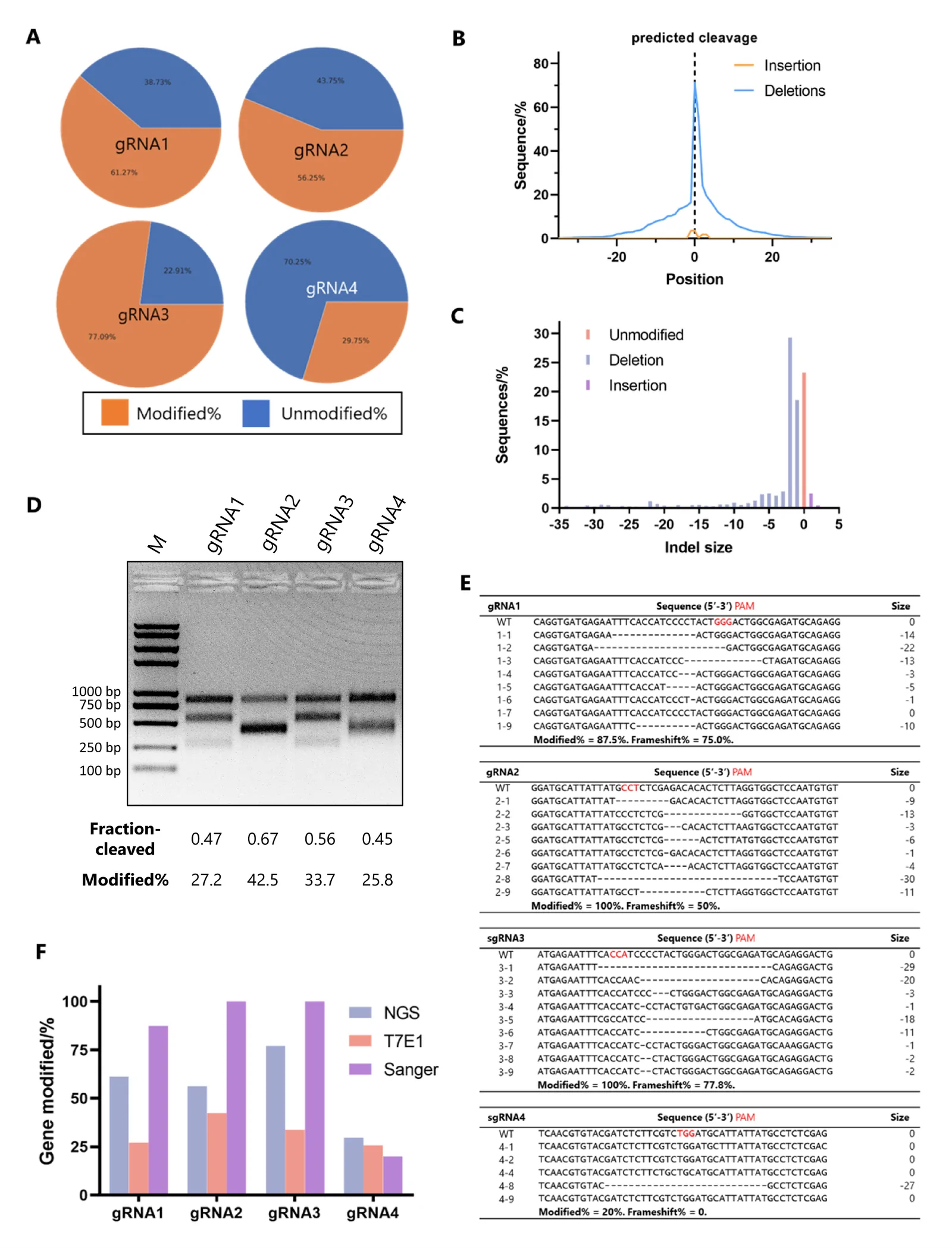
Validation of IndelQuant in *TYR*-edited DF-1 cells. (**A**) Editing efficiencies of four TYR-targeting gRNAs quantified by IndelQuant. (**B**) Distribution of indel positions in a representative gRNA3-edited DF-1 cell sample. (**C**) Distribution of indel sizes in a representative gRNA3-edited DF-1 cell sample. (**D**) T7 endonuclease I assay for detecting CRISPR-induced indels in DF-1 cells, with editing efficiencies estimated from gel band intensities. (**E**) Topo-clone-based Sanger sequencing analysis of TYR-edited DF-1 cells. Individual bacterial colonies were sequenced to identify indel-containing alleles and frameshift variants. Red characters indicate the protospacer adjacent motif (PAM). (**F**) Comparison of editing-efficiency estimates obtained using IndelQuant, the T7 endonuclease I assay, and topo-clone-based Sanger sequencing. IndelQuant provides read-level quantification together with graphical indel-position and indel-size profiles.

We first evaluated IndelQuant inDF-1 cells edited using four *TYR*-targeting gRNAs (Supplementary Table S2). Sequencing outputs and barcode assignments are summarized in Supplementary Table S3. Nearly all reads mapped to the target locus (Supplementary Tables S5 and S6). IndelQuant estimated editing efficiencies of 61.27%, 56.25%, 77.09%, and 29.75% for gRNA1–4, respectively (Figure 2A). Graphical mutation profiles showed that indels clustered near the predicted gRNA cleavage sites, with predominant indel sizes ranging from −5 to +1 bp (Figure 2B,C). These findings show that IndelQuant can quantify editing efficiency while resolving individual mutation patterns.

We also assessed the edited samples using conventional assays. The T7E1 assay detected editing in all gRNA groups, with estimated efficiencies of 27.2%, 42.5%, 33.7%, and 25.8% (Figure 2D). Clone-based Sanger sequencing also confirmed the presence of indels in the edited groups; however, because only a limited number of colonies were analyzed, the results were not treated as a precise quantitative reference (Figure 2E). All three methods identified the same edited groups, although the level of information they provided differed. T7E1 provided gel-based estimates, whereas clone-based Sanger sequencing sampled a relatively small number of DNA molecules. IndelQuant provided read-level quantification together with indel-position and indel-size profiles, offering greater detail for heterogeneous samples (Figure 2F).

### 2.3. Application of IndelQuant to Genome-Edited Chickens

**Figure 3:**
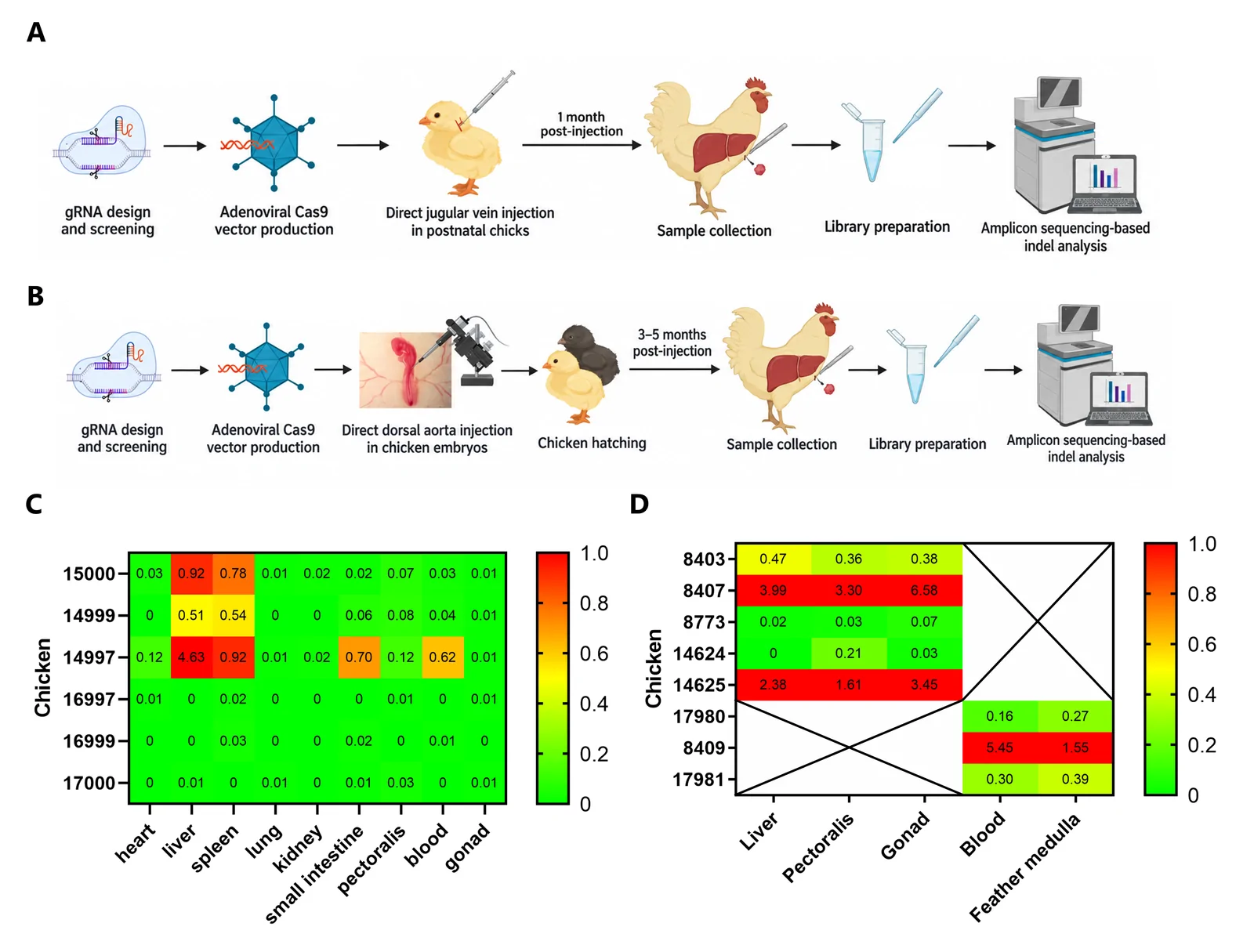
Application of IndelQuant to tissue-level indel analysis in genome-edited chickens. (**A**,**B**) Schematic overview of the two adenoviral CRISPR-Cas9 delivery strategies used to generate TYR-edited chickens: postnatal jugular-vein injection and embryonic dorsal-aorta injection; (**C**) Indel frequencies observed in tissues from chickens generated by postnatal jugular-vein delivery of Ad5-Cas9/gRNA3. Non-targeting controls showed background indel read frequencies of 0–0.03%. This range represents the observed control background, not a validated limit of detection, limit of quantification, or sample-level false-positive rate. Values above 1% are shown in red; (**D**) Indel frequencies observed in tissues from chickens generated by embryonic dorsal-aorta delivery of Ad5-Cas9/gRNA3. IndelQuant showed variation in indel read frequencies among the sampled tissues and animals. Values above 1% are shown in red.

IndelQuant was then applied to TYR-edited chimeric chickens generated using two adenoviral CRISPR-Cas9 delivery strategies (Figure 3A,B; Supplementary Figure S6). Sequencing details are summarized in Supplementary Table S5, and sample-specific barcode primer assignments are listed in Supplementary Tables S7 and S8.

In the postnatal delivery experiment, observed indel frequencies in non-targeting controls ranged from 0 to 0.03% across tissues. These values represent observed background indel read frequencies rather than a sample-level false-positive rate. Limits of detection and quantification were not established in this study. Accordingly, values within or near this range were interpreted cautiously, while higher values were reported descriptively without treating 0.03% as a validated detection threshold. Against this background, *TYR*-targeted chickens showed variable indel frequencies, with higher values in the liver and spleen samples from selected animals (Figure 3C). For embryonic delivery, adenoviral CRISPR-Cas9 vectors were injected into embryonic dorsal aorta. Eight chickens hatched (Supplementary Figure S7), and tissues were collected from both deceased and living animals. IndelQuant revealed differences in indel read frequencies among tissues and animals. For example, chickens 8407, 14,625, and 8409 showed relatively high indel read frequencies in specific tissues (Figure 3D). These findings illustrate how IndelQuant can summarize tissue-level indel frequencies across multiple chickens while preserving the underlying mutation sequences for inspection.

### 2.4. Literature-Based Comparison of CRISPR-Induced Indel Detection Methods

**Figure 4:**
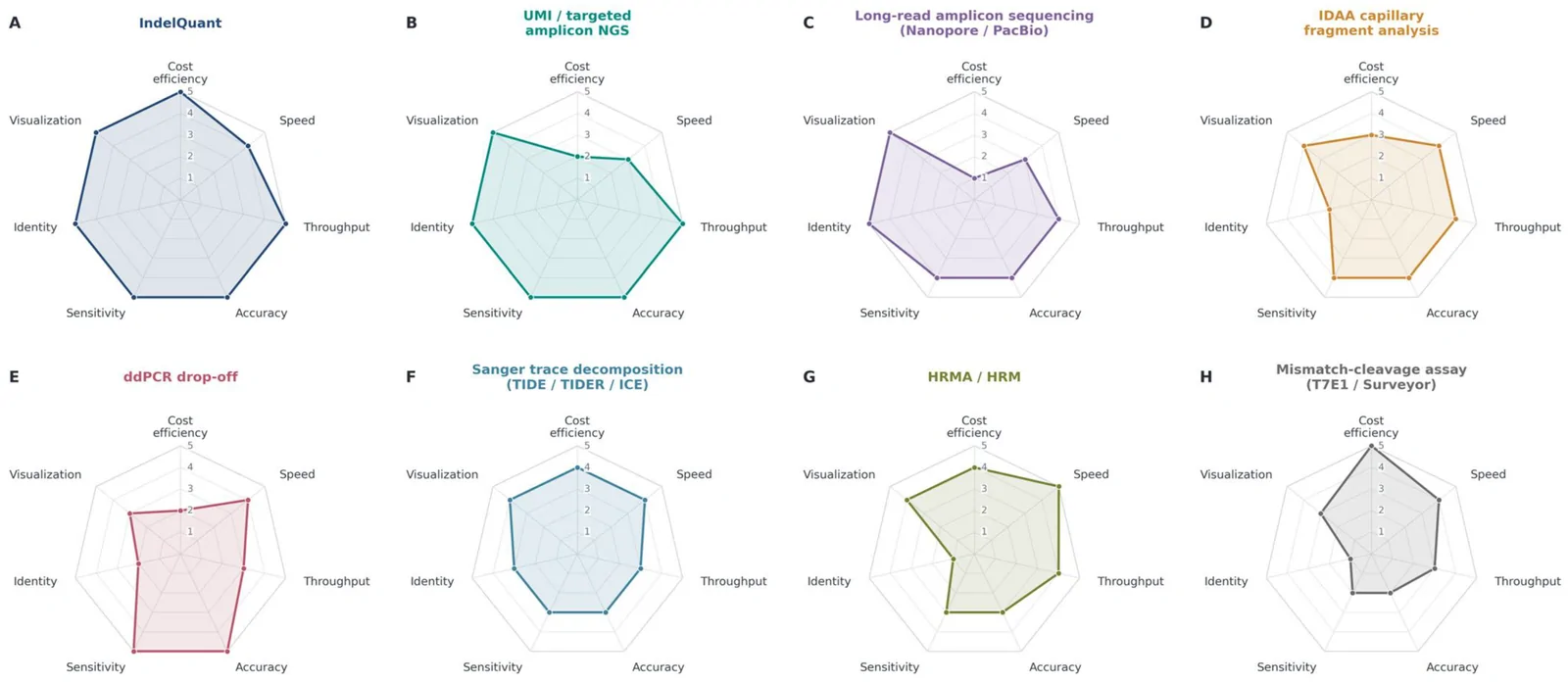
Multidimensional comparison of representative methods for CRISPR-induced indel detection. Radar plots compare eight methods using seven evaluation criteria: cost efficiency, speed, throughput, accuracy, sensitivity, allele identification (Identity), and visualization. (**A**) IndelQuant. (**B**) UMI/targeted amplicon next-generation sequencing (NGS). (**C**) Targeted long-read amplicon sequencing using Nanopore or PacBio platforms. (**D**) IDAA capillary fragment analysis. (**E**) Droplet digital PCR (ddPCR) drop-off assays. (**F**) Sanger trace decomposition using TIDE, TIDER, or ICE. (**G**) High-resolution melting analysis (HRMA/HRM). (**H**) Mismatch-cleavage assays using T7E1 or Surveyor. Scores of 1–5 are reproduced from Supplementary Table S10. Scores reflect qualitative assessments rather than measurements obtained from a standardized comparative experiment.

Within the predefined scoring framework, sequencing-based approaches generally received higher weighted scores for applications requiring exact indel identification and quantitative allele profiles (Figure 4; Supplementary Tables S9–S11). These scores summarize method characteristics under the selected criteria and weights; they should not be interpreted as experimentally established differences in accuracy, sensitivity, or overall analytical performance.

IndelQuant achieved the highest weighted score (97.5/100), reflecting the framework’s emphasis on per-sample cost, batch processing, throughput, sequence-level information, and integrated visualization. UMI- or molecular-barcoded-based targeted amplicon sequencing [30] received 87.5/100, balancing the value of molecule-level consensus against additional library preparation complexity and cost. Targeted long-read amplicon sequencing [31] received a score of 75.0/100, reflecting its ability to resolve large deletions, complex rearrangements, and long haplotypes that short-read assays may miss.

Among non-NGS approaches, IDAA [32,33] and ddPCR [34,35] received 71.5/100 and 71.0/100, respectively. IDAA provided rapid, quantitative fragment-size profiles, whereas ddPCR supports targeted quantification of known alleles or probe drop-off events. Sanger trace decomposition methods, HRM, and qPCR-based assays [36] were useful mid-tier approaches for rapid and relatively inexpensive screening, but their limited ability to resolve allele identities lowered their overall ranking. Low-cost endpoint assays, including T7E1/Surveyor/EMC and RFLP [23,37] or fragment-length assays, remained appropriate for initial screening but scored lowest because they are semiquantitative and do not define the edited alleles. Genome-wide off-target discovery methods also received moderate scores because their primary purpose differs from the on-target indel analysis emphasized here.

Because these rankings reflect the selected criteria and weights, they are specific to the intended application and may change when priorities differ. Under this framework, IndelQuant emerges as a practical option for routine, multi-sample indel profiling, alongside screening approaches such as HRM, TIDE/ICE [17,38], qPCR, and IDAA. UMI-based sequencing, long-read sequencing, and genome-wide discovery remain complementary options when rare-variant analysis, structural complexity, or off-target assessment is the primary objective.

## 3. Discussion

Although PGC-mediated approaches and viral CRISPR delivery systems have made avian genome editing increasingly feasible, detecting low-frequency editing signals across multiple tissues remains a major challenge.. An effective screening method for poultry genome editing should therefore be affordable, sensitive to low-frequency signals, capable of resolving sequence variants, and straightforward to interpret. In this study, we developed IndelQuant as a graphical software package and workflow for low-cost amplicon sequencing analysis of genome-edited chickens. The method integrates sample-specific barcode PCR, pooled amplicon sequencing, AdapterRemoval3-based demultiplexing, CRISPResso2-based indel quantification, and graphical mutation profiling.

The potential cost savings offered by IndelQuant arise from barcode-based multiplexing. By incorporating sample-specific barcode pairs into target-specific primers, multiple samples can be pooled into one sequencing library while retaining sample identity. This design simplifies library preparation burden and makes targeted amplicon sequencing more practical for routine screening. Similar barcode-based NGS strategies have previously been shown to support high-throughput CRISPR mutation screening [29]. IndelQuant extends this concept into a user-oriented workflow for animal genome-editing analysis by combining pooled sequencing with automated demultiplexing, CRISPResso2 configuration, output monitoring, visualization, and CSV export. Importantly, IndelQuant retains a key strength of NGS-based indel detection: accuracy and sequence-level resolution.

Several limitations warrant consideration. First, IndelQuant is designed for targeted amplicon sequencing and is not a genome-wide off-target detection method. Second, short-read amplicon sequencing may miss very long indels or large structural variants extending beyond the amplified region. Third, sequencing turnaround time remains dependent on sample submission and sequencing-service availability. Fourth, the workflow should be evaluated with appropriate negative controls before routine use with new species, loci, barcode combinations, or sequencing batches.

In summary, IndelQuant provides a low-cost graphical workflow for routine indel analysis in genome-edited chickens. It is particularly useful for screening multiple samples for low-frequency and mosaic indels while retaining sequence-level mutation information. With appropriate validation, the same workflow could be adapted to other genome-edited animal systems.

## 4. Materials and Methods

### 4.1. Animals

Freshly laid eggs from the Beijing You and Xinyang Black chicken were incubated in an Ova-Easy Advance Series II incubator (Brinsea, Titusville, FL, USA) at 37.8 °C and 60% relative humidity. Chickens were raised at the Animal Husbandry and Veterinary Research Institute, Shanghai Academy of Agricultural Science, Shanghai, China. The ages, sexes, breeds, and experimental assignments of the chickens are listed in Supplementary Table S1.

### 4.2. Cell Culture

DF-1 cells (ATCC CRL-12203) were purchased from the National Collection of Authenticated Cell Culture (Shanghai, China) and maintained in Dulbecco’s Modified Eagle Medium (Gibco, Grand Island, NY, USA) supplemented with 10% (*v*/*v*) fetal bovine serum (Gibco, USA) at 37 °C under 5% CO_2_.

### 4.3. Guide RNA Design and Plasmid Construction

Guide RNAs (gRNAs) targeting the chicken *TYR* gene (NCBI Gene ID: 373971; GRCg6a) were designed using CHOPCHOP v3 [39]. The gRNAs and synthesized oligonucleotides used are listed in Supplementary Table S2. PSpCas9(BB)-2A-Puro (PX459) V2.0 (Addgene #62988) was a gift from Feng Zhang. Annealed gRNA oligonucleotides were cloned into the *Bbs*I-digested PX459 to generate Cas9/gRNA plasmids.

### 4.4. Adenoviral Vector Production

The adenoviral Cas9 vector pAV[CRISPR]-hCas9:P2A:EGFP-U6>gRNA3 (Ad5-Cas9/gRNA3), co-expressing SpCas9, gRNA-TYR3 and EGFP, and the control vector pAV[Exp]-CBh>hCas9(ns):P2A:EGFP (Ad5-Cas9/ns), co-expressing spCas9 and EGFP without a targeting gRNA, were obtained from VectorBuilder (Chicago, IL, USA). The structures of the recombinant adenoviral vectors are shown in Supplementary Figure S6. The final titers of Ad5-Cas9/gRNA3 and Ad5-Cas9/ns were 1.63 × 10^12^ and 1.83 × 10^12^ viral particles (VP)/mL, respectively.

### 4.5. Cell Transfection

DF-1 cells were seeded in six-well plates (Corning, Corning, NY, USA) at 2 × 10^5^ cells per well. After 24 h, cells in each well were transfected with 2.5 μg of Cas9/gRNA plasmids using Lipofectamine 3000 (Thermo Fisher Scientific, Waltham, MA, USA) according to the manufacturer’s instructions. Puromycin selection was initiated 48 h after transfection by supplementing the complete medium with puromycin at a final concentration of 2 μg/mL.

### 4.6. Adenoviral Vector Delivery

For postnatal *in vivo* delivery, 100 μL of Ad5-Cas9/gRNA3 was injected directly into the jugular vein of postnatal chicks using Syringes (Hamilton, NV, USA). For *in ovo* delivery, the adenoviral vector injection was performed as previously described, with minor modifications [7]. In brief, eggs were incubated for 50 h to reach Hamburger–Hamilton (HH) stage 14. A small opening was then made at the blunt end of the eggshell, and 2–4 µL of Ad5-Cas9/gRNA3 was injected into the dorsal aorta of the chicken embryo using a CellTram 4 oil microinjector (Eppendorf, Hamburg, Germany). The openings were then sealed with adhesive tape, and the eggs were incubated with their blunt end facing upward. Eggs were kept on rotating trays for 16 d before being transferred to hatching trays until hatching. Ad5-Cas9/ns was used as the control.

### 4.7. Genomic DNA Preparation

Cells were harvested after one week of puromycin selection. For *in ovo* experiments, liver, pectoralis muscle, and gonad samples were collected from embryos or chicks that did not survive to sampling, whereas blood and feather medulla were collected from living chicks. For postnatal in vivo experiments, chickens were humanely euthanized 1 month after injection, and heart, liver, spleen, lung, kidney, small intestine, pectoralis muscle, blood, and gonad samples were collected. Genomic DNA was extracted using a Tissue DNA kit (Omega Bio-tek, Norcross, GA, USA) following the manufacturer’s instructions.

### 4.8. T7 Endonuclease I Assay

The mismatch cleavage assay was performed using T7 endonuclease I (New England Biolabs, Ipswich, MA, USA) following a previously established protocol [14,40]. In brief, the target region was amplified using the 2× Phanta Max Master Mix (Vazyme, Nanjing, China) with the primers TYR-F (5′-AGCAGAGTCAGTGGTGAAGC-3′) and TYR-E1-R (5′-TCTTGCCCTCCCCTTACCTT-3′). The PCR cycling conditions were 95 °C for 3 min; 36 cycles of 95 °C for 15 s, 58 °C for 20 s, and 72 °C for 20 s; and 72 °C for 5 min. PCR products were purified using a Cycle pure kit (Omega Bio-tek, GA, USA), denatured and reannealed using the following temperature program: 95 °C for 5 min, −2 °C/min to 85 °C, and −0.1 °C/min to 25 °C. The reannealed products were digested with 1 μL of T7E1 in a 20 μL reaction at 37 °C for 30 min. The products were analyzed on 2% agarose gels, and the gene-editing efficiency was estimated using ImageJ v1.8.0.

### 4.9. Topo-Clone-Based Sanger Sequencing

PCR products generated for the T7E1 assay were cloned into the pCE2-Topo vector using the TA/Blunt-Zero Cloning Kit (Vazyme, Nanjing, China) according to the manufacturer’s instructions. The resulting plasmids were transformed into DH5α and the transformed bacteria were then plated on lysogeny broth agar containing 100 mg/L ampicillin. For each gRNA group, five to nine sequence-verified colonies were included in the clone-based Sanger analysis, depending on colony recovery and sequence quality. Sequences were aligned with MUSCLE in MEGA v11.0.10 to identify indels.

### 4.10. IndelQuant Workflow Design

IndelQuant was designed as a modular, low-cost, graphical workflow for the analysis of CRISPR-induced indels from pooled amplicon sequencing data. The workflow comprises five operational modules: sample collection and genomic DNA extraction, target-specific PCR with sample-specific barcode pairs, pooled amplicon library construction and paired-end sequencing, barcode-based demultiplexing and indel quantification, and graphical output of editing efficiency and mutation profiles (Figure 1). The software was designed to minimize manual intervention of the pipeline by integrating AdapterRemoval3 configuration, CRISPResso2 parameter setup, dependency and environment checking, automated output monitoring, result parsing, visualization, and data export into one interface.

### 4.11. Sample-Specific Barcode Design and Library Preparation

Target regions were amplified from genomic DNA using the 2× Phanta Max Master Mix (Vazyme, Nanjing, China). Sample-specific barcode pairs were attached to the 5′ ends of both forward and reverse primers, allowing multiple samples to be pooled into a single amplicon library while retaining sample identity (Supplementary Tables S3 and S4). PCR conditions were 95 °C for 3 min; 36 cycles of 95 °C for 15 s, 58 °C for 15 s, and 72 °C for 20 s; and 72 °C for 5 min. The amplicons were pooled and purified using the Cycle Pure Kit (Omega Bio-tek, GA, USA). Amplicon libraries were prepared using the DNA PCR-Free Library Prep Kit (Illumina, San Diego, CA, USA) according to the manufacturer’s instructions and quantified by agarose gel electrophoresis and quantitative PCR.

### 4.12. Amplicon Sequencing

Amplicon libraries were sequenced on the NovaSeq 6000 platform (Illumina, CA, USA) in PE250 or PE150 mode by Novogene(Beijing, China). The target sequencing depth was approximately one million reads per library. Sequencing outputs and quality metrics are summarized in Supplementary Tables S5 and S6.

### 4.13. IndelQuant Data Processing and Graphical Output

With IndelQuant, raw paired-end reads were first processed using AdapterRemoval v3.0.0 and sample-specific barcode pairs. AdapterRemoval3 performed barcode-based demultiplexing, removal of unassigned or unpaired reads, quality filtering, sequence trimming, and paired-end read joining. The processed reads were then analyzed using CRISPResso2 v2.3.4 for reference-guided mapping and indel quantification with the following parameters: *--trim_sequences --trimmomatic_options_string slidingwindow:4:10 --ignore_substitutions --plot_histogram_outliers --min_frequency_alleles_around_cut_to_plot 0.01*. During analysis, IndelQuant monitors CRISPResso2 output folders, identifies valid result files, parses editing statistics, and generates graphical summaries of editing efficiency, indel-position distribution, indel-size distribution, and sample-level mutation profiles. Summary results can be exported in CSV format. The software is available at https://github.com/rexhan6/IndelQuant.

### 4.14. Computing Environment and Benchmark

IndelQuant was implemented in Python and tested with Python 3.13.13 on Windows 10 22H2. The graphical interface was implemented in CustomTkinter, with NumPy, pandas, Matplotlib, and openpyxl supporting data processing, visualization, and file export. The analysis workflow integrates AdapterRemoval v3.0.0-38e6a81 and CRISPResso2 v2.3.4. Dependencies were installed and configured using Conda and the software’s environment setup interface. Before analysis, IndelQuant can install the required analysis environment and automatically verify their availability. Memory usage and runtime were evaluated using the test dataset; the hardware specifications, dataset size, processing settings, memory usage, and runtime benchmarks are reported in the Supplementary Table S12. These measurements describe resource use under the tested conditions rather than a universal minimum hardware requirement.

### 4.15. Comparative Scoring of Indel Detection Methods

We conducted a literature-based comparison of methods for detecting and quantifying CRISPR-induced indels. Candidate methods were selected a priori from established screening, sequencing, PCR-based, capillary electrophoresis, digital PCR, long-read sequencing, and genome-wide off-target discovery workflows. The methods evaluated included mismatch-cleavage assays (T7E1, Surveyor, and related endonuclease mismatch cleavage assays), RFLP/PCR fragment-length and indCAPS assays, high-resolution melting analysis, Sanger sequencing with manual review or amplicon cloning, Sanger trace decomposition methods (TIDE, TIDER, and ICE), indel detection by amplicon analysis (IDAA), IndelQuant as an in-house low-cost barcoded amplicon NGS workflow, UMI or molecular-barcoded targeted amplicon sequencing, droplet digital PCR, qPCR-based assays, targeted long-read amplicon sequencing, and genome-wide DSB/off-target discovery methods such as GUIDE-seq, SITE-seq, CIRCLE-seq, and CHANGE-seq. Primary research papers, benchmarking studies, protocols, and representative CRISPR application reports were identified through title- and keyword-based searches of CrossRef, PubMed, and other scholarly databases. Each method was evaluated across seven operational criteria: low cost, speed, throughput, accuracy, sensitivity, allele or event identity, and visualization. Each dimension was scored from 1 to 5, with higher scores indicating more favorable characteristics under the selected framework (Supplementary Table S9). IndelQuant’s cost-efficiency score was assigned for the intended in-house deployment model, where sample barcoding, batching, and automated local analysis substantially reduce marginal per-sample cost. The overall score was calculated as a weighted average of the following criteria: low cost: 12.5%, speed: 12.5%, throughput: 15%, accuracy: 20%, sensitivity: 15%, identity: 15%, and visualization: 10%. Accuracy, sensitivity, and identity were weighted more heavily because these features are particularly important for detailed indel characterization rather than preliminary screening alone.

### 4.16. Statistical Analysis

This study was designed primarily as workflow development and descriptive methodological evaluation; therefore, no inferential comparisons among delivery routes, tissues, or assay platforms were intended. For DF-1 cell experiments, each gRNA-transfected cell population and its corresponding sequencing library were analyzed as a single pooled analytical sample. Accordingly, editing estimates obtained from IndelQuant, the T7E1 assay, and clone-based Sanger sequencing were interpreted descriptively without formal statistical comparison. For postnatal chicken delivery, the animal was considered the experimental unit, whereas tissues represented repeated sample types within the same animal and were not treated as independent biological replicates. Chickens receiving non-targeting Ad5-Cas9/ns served as background controls. For embryonic delivery, each sampled chicken was treated as one experimental unit, and tissue-level values were interpreted descriptively as mosaic editing profiles. Editing frequencies for individual chickens and tissues were calculated as Modified reads divided by Reads_aligned. No statistical models were fitted, and no hypothesis tests were performed.

## 5. Patents

The authors declare that a patent related to this manuscript has been filed.

## Data Availability

The IndelQuant v0.1.1 is publicly available at https://github.com/rexhan6/IndelQuant. The raw sequence data reported in this paper have been deposited in the Genome Sequence Archive in the National Genomics Data Center, China National Center for Bioinformation/Beijing Institute of Genomics, Chinese Academy of Sciences, under accession CRA044532, which is publicly accessible at https://ngdc.cncb.ac.cn/gsa.

## Supplementary Materials

The following supporting information can be downloaded at https://www.mdpi.com/article/10.3390/ijms27209054/s1.

## Abbreviations

The following abbreviations are used in this manuscript:

Ad5	Adenovirus serotype 5
CRISPR	Clustered regularly interspaced short palindromic repeats
gRNA	Guide RNA
GUI	Graphical user interface
indel	Insertion and deletion mutation
NGS	Next-generation sequencing
TYR	Tyrosinase

## References

[1] Park J.S., Lee K.Y., Han J.Y. (2020). Precise Genome Editing in Poultry and Its Application to Industries. Genes.

[2] Van De Lavoir M.C., Diamond J.H., Leighton P.A., Mather-Love C., Heyer B.S., Bradshaw R., Kerchner A., Hooi L.T., Gessaro T.M., Swanberg S.E. (2006). Germline Transmission of Genetically Modified Primordial Germ Cells. Nature.

[3] Véron N., Qu Z., Kipen P.A.S., Hirst C.E., Marcelle C. (2015). CRISPR Mediated Somatic Cell Genome Engineering in the Chicken. Dev. Biol..

[4] Kim Y.-M., Woo S.-J., Han J.-Y. (2023). Strategies for the Generation of Gene Modified Avian Models: Advancement in Avian Germline Transmission, Genome Editing, and Applications. Genes.

[5] Qin C., Jiang S., Xu K., Zhu J., Wang L., Yang W., Xiao F., Yang K., Huang Q., Meng H. (2024). One-Step Genetic Modification by Embryonic Doral Aorta Injection of Adenoviral CRISPR/Cas9 Vector in Chicken. Int. J. Mol. Sci..

[6] Tabebordbar M., Zhu K., Cheng J.K.W., Chew W.L., Widrick J.J., Yan W.X., Maesner C., Wu E.Y., Xiao R., Ran F.A. (2016). In Vivo Gene Editing in Dystrophic Mouse Muscle and Muscle Stem Cells. Science.

[7] Lee J., Ma J., Lee K. (2019). Direct Delivery of Adenoviral CRISPR/Cas9 Vector into the Blastoderm for Generation of Targeted Gene Knockout in Quail. Proc. Natl. Acad. Sci. USA.

[8] Oishi I., Yoshii K., Miyahara D., Kagami H., Tagami T. (2016). Targeted Mutagenesis in Chicken Using CRISPR/Cas9 System. Sci. Rep..

[9] Chojnacka-Puchta L., Sawicka D. (2020). CRISPR/Cas9 Gene Editing in a Chicken Model: Current Approaches and Applications. J. Appl. Genet..

[10] Woodcock M.E., Idoko-Akoh A., McGrew M.J. (2017). Gene Editing in Birds Takes Flight. Mamm. Genome.

[11] Sid H., Schusser B. (2018). Applications of Gene Editing in Chickens: A New Era Is on the Horizon. Front. Genet..

[12] He J., Shi N., Yao H., Li J., Wang Y., Zhang J. (2025). Genome Editing in the Chicken: From PGC-Mediated Germline Transmission to Advanced Applications. Int. J. Mol. Sci..

[13] Huang M.C., Cheong W.C., Lim L.S., Li M.-H. (2012). A Simple, High Sensitivity Mutation Screening Using Ampligase Mediated T7 Endonuclease I and Surveyor Nuclease with Microfluidic Capillary Electrophoresis. Electrophoresis.

[14] Guschin D.Y., Waite A.J., Katibah G.E., Miller J.C., Holmes M.C., Rebar E.J. (2010). A Rapid and General Assay for Monitoring Endogenous Gene Modification. Engineered Zinc Finger Proteins: Methods and Protocols.

[15] Sentmanat M.F., Peters S.T., Florian C.P., Connelly J.P., Pruett-Miller S.M. (2018). A Survey of Validation Strategies for CRISPR-Cas9 Editing. Sci. Rep..

[16] Bennett E.P., Petersen B.L., Johansen I.E., Niu Y., Yang Z., Chamberlain C.A., Met Ö., Wandall H.H., Frödin M. (2020). INDEL Detection, the ‘Achilles Heel’ of Precise Genome Editing: A Survey of Methods for Accurate Profiling of Gene Editing Induced Indels. Nucleic Acids Res..

[17] Brinkman E.K., Chen T., Amendola M., van Steensel B. (2014). Easy Quantitative Assessment of Genome Editing by Sequence Trace Decomposition. Nucleic Acids Res..

[18] Ran F.A., Hsu P.D., Wright J., Agarwala V., Scott D.A., Zhang F. (2013). Genome Engineering Using the CRISPR-Cas9 System. Nat. Protoc..

[19] Taheri-Ghahfarokhi A., Taylor B.J.M., Nitsch R., Lundin A., Cavallo A.-L., Madeyski-Bengtson K., Karlsson F., Clausen M., Hicks R., Mayr L.M. (2018). Decoding Non-Random Mutational Signatures at Cas9 Targeted Sites. Nucleic Acids Res..

[20] Hendel A., Fine E.J., Bao G., Porteus M.H. (2015). Quantifying On- and off-Target Genome Editing. Trends Biotechnol..

[21] Chen S., Oikonomou G., Chiu C.N., Niles B.J., Liu J., Lee D.A., Antoshechkin I., Prober D.A. (2013). A Large-Scale in Vivo Analysis Reveals That TALENs Are Significantly More Mutagenic than ZFNs Generated Using Context-Dependent Assembly. Nucleic Acids Res..

[22] Clement K., Rees H., Canver M.C., Gehrke J.M., Farouni R., Hsu J.Y., Cole M.A., Liu D.R., Joung J.K., Bauer D.E. (2019). CRISPResso2 Provides Accurate and Rapid Genome Editing Sequence Analysis. Nat. Biotechnol..

[23] Gong Z., Zhang Y., Xia D., Yoon S., Crisp P.A., Botella J.R. (2025). Comprehensive Benchmarking of Genome Editing Quantification Methods for Plant Applications. iScience.

[24] Yao B., Yang Q., Gonçalves M.A.F.V., Schiffelers R., Sluijter J.P.G., Lei Z. (2025). Comparative Analysis of Methods for Assessing On-Target Gene Editing Efficiencies. Methods Protoc..

[25] Zou K., Wang F., Zhang Z., Zhou Y., Li P., Wang D., Zhu M., Jia C., Wei Z. (2023). Optimized CRISPR/Cas9 System for Gene Knockout in Chicken DF1 Cells. Poult. Sci..

[26] Brockman Q.R., Scherer A., McGivney G.R., Gutierrez W.R., Rytlewski J., Sheehan A., Warrier A., Laverty E.A., Roughton G., Carnevale N.C. (2023). Discrepancies in Indel Software Resolution with Somatic CRISPR/Cas9 Tumorigenesis Models. Sci. Rep..

[27] Sanvicente-García M., García-Valiente A., Jouide S., Jaraba-Wallace J., Bautista E., Escobosa M., Sánchez-Mejías A., Güell M. (2023). CRISPR-Analytics (CRISPR-a): A Platform for Precise Analytics and Simulations for Gene Editing. PLoS Comput. Biol..

[28] Schubert M., Lindgreen S., Orlando L. (2016). AdapterRemoval v2: Rapid Adapter Trimming, Identification, and Read Merging. BMC Res. Notes.

[29] Bell C.C., Magor G.W., Gillinder K.R., Perkins A.C. (2014). A High-Throughput Screening Strategy for Detecting CRISPR-Cas9 Induced Mutations Using next-Generation Sequencing. BMC Genom..

[30] Carrington B., Bishop K., Sood R. (2022). A Comprehensive Review of Indel Detection Methods for Identification of Zebrafish Knockout Mutants Generated by Genome-Editing Nucleases. Genes.

[31] McFarlane G.R., Polanco J.V.C., Bogema D. (2024). CRISPR-Cas Guide RNA Indel Analysis Using CRISPResso2 with Nanopore Sequencing Data. BMC Res. Notes.

[32] Lonowski L.A., Narimatsu Y., Riaz A., Delay C.E., Yang Z., Niola F., Duda K., Ober E.A., Clausen H., Wandall H.H. (2017). Genome Editing Using FACS Enrichment of Nuclease-Expressing Cells and Indel Detection by Amplicon Analysis. Nat. Protoc..

[33] Yang X. (2015). Applications of CRISPR-Cas9 Mediated Genome Engineering. Mil. Med. Res..

[34] Miyaoka Y., Berman J.R., Cooper S.B., Mayerl S.J., Chan A.H., Zhang B., Karlin-Neumann G.A., Conklin B.R. (2016). Systematic Quantification of HDR and NHEJ Reveals Effects of Locus, Nuclease, and Cell Type on Genome-Editing. Sci. Rep..

[35] Mock U., Hauber I., Fehse B. (2016). Digital PCR to Assess Gene-Editing Frequencies (GEF-dPCR) Mediated by Designer Nucleases. Nat. Protoc..

[36] Dabrowska M., Czubak K., Juzwa W., Krzyzosiak W.J., Olejniczak M., Kozlowski P. (2018). qEva-CRISPR: A Method for Quantitative Evaluation of CRISPR/Cas-Mediated Genome Editing in Target and off-Target Sites. Nucleic Acids Res..

[37] Vouillot L., Thélie A., Pollet N. (2015). Comparison of T7E1 and Surveyor Mismatch Cleavage Assays to Detect Mutations Triggered by Engineered Nucleases. G3.

[38] Conant D., Hsiau T., Rossi N., Oki J., Maures T., Waite K., Yang J., Joshi S., Kelso R., Holden K. (2022). Inference of CRISPR Edits from Sanger Trace Data. CRISPR J..

[39] Labun K., Montague T.G., Krause M., Torres Cleuren Y.N., Tjeldnes H., Valen E. (2019). CHOPCHOP v3: Expanding the CRISPR Web Toolbox beyond Genome Editing. Nucleic Acids Res..

[40] Mashal R.D., Koontz J., Sklar J. (1995). Detection of Mutations by Cleavage of DNA Heteroduplexes with Bacteriophage Resolvases. Nat. Genet..

