# Supplementary material for: IndelQuant: A Workflow for Cost-Efficient Amplicon Sequencing Analysis of Genome-Edited Chickens
Source: Int J Mol Sci. 2026 Oct 13;27(20):9054. doi: 10.3390/ijms27209054 (PMC13705014; doi:10.3390/ijms27209054)
Supplement: Supplementary file 1 [file ijms-27-09054-s001.zip › ijms-4519078-supplementary.pdf]

Supplementary materials

IndelQuant: a workflow for cost-efficient amplicon sequencing analysis of genome-edited chickens

Supplementary Tables S1–S12 and Supplementary Figures S1–S7.

Supplementary Table S1. Experimental assignments of the chickens.

No	Wing band	Sex	Denote	Breed	Born date
1	15000	F	T1	Beijing You	26 Aug. 2021
2	14999	M	T2		
3	14997	M	T3		
4	16997	F	CT1		
5	16999	M	CT2		
6	17000	F	CT3		
7	17980	F	T9		19 June 2020
8	17981	F	T10		
9	8409	F	T11		3 Aug. 2020
10	8403	F	T4		
11	8407	F	T5		
12	8773	M	T6	Xinyang Black	22 Aug 2020
13	14624	M	T7		
14	14625	F	T8		

Supplementary Table S2. Synthetic oligonucleotides for gRNA synthesis used in this study.

Denote	gRNA sequence	Oligo name	Sequence (5'−3')
gRNA1	GAATTTCACCATCC CCTACT	cTYR.e1.S1A	CACCGGAATTTCACCATCCCCTACT
		cTYR.e1.S1B	AAACAGTAGGGGATGGTGAAATTCC
gRNA2	CTCGAGACACACTC TTAGGT	cTYR.e1.S2A	CACCGACCTAAGAGTGTGTCTCGAG
		cTYR.e1.S2B	AAACCTCGAGACACACTCTTAGGTC
gRNA3	TCCCCTACTGGGAC TGGCGA	cTYR.e1.S3A	CACCGTCGCCAGTCCCAGTAGGGGA
		cTYR.e1.S3B	AAACTCCCCTACTGGGACTGGCGAC
gRNA4	ACGTGTACGATCTC TTCGTC	cTYR.e1.S4A	CACCGACGTGTACGATCTCTTCGTC
		cTYR.e1.S4B	AAACGACGAAGAGATCGTACACGTC

Supplementary Table S3. Barcode sequences and sample-specific assignments used in *TYR*-edited DF-1 cells library construction.

gRNAs	Barcoded forward primer (5'−3')	Reverse primer (5'−3')
gRNA1	AGACTCTGATTTTGCCCATGAAGCCCC	TTTGCCTGTCCTTACCTGCC
gRNA2	AGTCACAGCTCCAACCCCATGTTCAGA	ACACAGTCCTCTGCATCTCG
gRNA3	TCAGAGAGATTTTGCCCATGAAGCCCC	TTTGCCTGTCCTTACCTGCC
gRNA4	TGACTGAGCTCCAACCCCATGTTCAGA	ACACAGTCCTCTGCATCTCG

Supplementary Table S4. Barcode sequences and sample-specific assignments used in *TYR*-edited chicken tissues library construction.

Type	Abbr.	Oligo name	Sequence (5'–3')
Barcoded forward primer	A	TYRe1NovaA	ATCACGTGCTCAGATGAACAACGGCT
	B	TYRe1NovaB	CGATGTTGCTCAGATGAACAACGGCT
	C	TYRe1NovaC	TTAGGCTGCTCAGATGAACAACGGCT
	D	TYRe1NovaD	TGACCATGCTCAGATGAACAACGGCT
	E	TYRe1NovaE	ACAGTGTGCTCAGATGAACAACGGCT
	F	TYRe1NovaF	GCCAATTGCTCAGATGAACAACGGCT
Barcoded reverse primer	1	TYRe1Nova1	CAGATCTGCCCTCCCCTTACCTTCAT
	2	TYRe1Nova2	ACTTGATGCCCTCCCCTTACCTTCAT
	3	TYRe1Nova3	GATCAGTGCCCTCCCCTTACCTTCAT
	4	TYRe1Nova4	TAGCTTTGCCCTCCCCTTACCTTCAT
	5	TYRe1Nova5	GGCTACTGCCCTCCCCTTACCTTCAT
	6	TYRe1Nova6	CTTGTATGCCCTCCCCTTACCTTCAT

Supplementary Table S5. Sequencing output and quality metrics for barcoded amplicon libraries.

Library	Platform	Mode	Reads	Data / Gbase	Q30
RC	NovaSeq 6000	PE150	1,525,477	0.46	96.8%
R1		PE250	1,283,764	0.64	95.1%
R2		PE250	1,600,779	0.80	95.3%
RB		PE250	1,079,028	0.54	93.2%

Supplementary Table S6. Sequencing output, read mapping, and modified rate for barcoded amplicon libraries from *TYR*-edited DF-1 cells.

gRNA	Raw reads	Aligned reads	Modified reads	Modified%
gRNA1	342,823	342,502	209,853	61.27%
gRNA2	627,014	626,491	352,405	56.25%
gRNA3	247,814	247,534	190,835	77.09%
gRNA4	307,826	307,560	91,487	29.75%

Supplementary Table S7. Sample-specific barcode primer assignments for amplicon libraries R1 and R2 from *TYR*-edited chicken tissues.

Library	Wing band	Heart	Liver	Spleen	Lung	Kidney	Intestine	Pectoralis	Blood	Gonad
R1	15000	A1	A2	A3	A4	A5	B1	B2	B3	B4
	14999	C1	C2	C3	C4	C5	D1	D2	D3	D4
	14997	E1	E2	E3	E4	E5	F1	F2	F3	F4
R2	16997	A1	A2	A3	A4	A5	B1	B2	B3	B4
	16999	C1	C2	C3	C4	C5	D1	D2	D3	D4
	17000	E1	E2	E3	E4	E5	F1	F2	F3	F4

Supplementary Table S8. Sample-specific barcode primer assignments for amplicon library RB from *TYR*-edited chicken tissues.

Library	Wing band	Liver	Pectoralis	Blood	Gonad	Feather
RB	17980	N/A	N/A	B1	N/A	B2
	8409	N/A	N/A	B3	N/A	B4
	17981	N/A	N/A	B5	N/A	B6
	8403	C1	C2	N/A	C3	N/A
	8407	C4	C5	N/A	C6	N/A
	8773	D1	D2	N/A	D3	N/A
	14624	D4	D5	N/A	D6	N/A
	14625	E1	E2	N/A	E3	N/A

Supplementary Table S9. Scoring system of CRISPR-induced indel detection methods.

Dimension	Score 5 anchor	Score 3 anchor	Score 1 anchor
Cost	PCR/gel-like per-sample cost, no special instrument	Sanger/capillary/qPCR or moderate consumables	long-read/genome-wide sequencing or expensive platform
Speed	same-day, <6 h after DNA	1-3 days	usually >1 week or complex batching
Throughput	many samples/loci per run, plate or indexed NGS scale	dozens of samples	low parallelization
Accuracy	quantitative and concordant with sequencing/known standards	semi-quantitative or model-dependent	qualitative or strongly biased
Sensitivity	low-frequency alleles detectable, roughly <=1% with proper controls	roughly 5-10%	poor below high editing fractions
Identity	exact allele sequence / allele spectrum / large-event structure	inferred or size/known-allele-limited	no allele identity
Visualization	rich allele plots/ /electropherograms/QC	simple gel/curve/droplet view	mostly numeric Ct/endpoint

Supplementary Table S10. Scoring review of CRISPR-induced indel detection methods.

Rank	Method	Cost	Speed	Throughput	Accuracy	Sensitivity	Identity	Visualization	Overall /100	Best use	Main limitation
1	IndelQuant	5	4	5	5	5	5	5	97.5	routine definitive on-target indel quantification across many samples at low marginal cost	requires a validated in-house workflow, controls, and sufficient read depth
2	UMI / targeted amplicon NGS	2	3	5	5	5	5	5	87.5	ultra-low-frequency or high-confidence molecular consensus allele spectrum	cost/library complexity; PCR/UMI design matters
3	Long-read amplicon sequencing	1	3	4	4	4	5	5	75.0	large indels, long haplotypes, distant variants, complex alleles	higher per-sample cost/error model; depth and consensus strategy critical
4	IDAA capillary fragment analysis	3	4	4	4	4	2	4	71.5	fast quantitative size-profile screening when sequence identity is not required	fragment length only; same-size different-sequence alleles collapse
5	ddPCR drop-off	2	4	3	5	5	2	3	71.0	sensitive quantification of known allele, WT loss, or drop-off editing frequency	probe/assay-specific; limited discovery of unknown indels
6	Sanger trace decomposition: TIDE / TIDER / ICE	4	4	3	3	3	3	4	67.0	cheap first-pass quantification at one locus with clean bulk Sanger traces	deconvolution assumptions; limited complex/multiplex/low-frequency calls
7	HRMA / HRM	4	5	4	3	3	1	4	66.5	rapid plate-scale mutant screening before sequencing	detects melting shifts, not allele identity; false negatives possible
8	qPCR-based assays	4	5	4	3	4	1	2	65.5	rapid plate screening or target/off-target quantitative evaluation	assay-primer dependent; usually no indel sequence/allele spectrum

9	Genome-wide DSB/off-target discovery (GUIDE-seq / CIRCLE-seq)	1	2	3	4	5	3	4	64.5	unbiased off-target site discovery, safety profiling	not a low-cost on-target indel quantification assay; needs follow-up targeted validation
10	Sanger sequencing + amplicon cloning	3	3	2	3	2	4	4	59.0	confirming clones or dominant alleles when sample count is small	cloning/sampling bias; bulk trace is weak for mixed indels
11	RFLP / PCR fragment-length	5	5	2	2	2	2	3	57.0	very cheap genotyping when indel changes size or restriction site	locus/indel constrained; weak quantification and identity
12	Mismatch-cleavage assay (T7E1 / Surveyor)	5	4	3	2	2	1	3	54.5	quick low-cost "did editing happen?" screen	heteroduplex formation and nuclease bias; no allele identity; semi-quantitative

Supplementary Table S11. Estimated costs and time requirements for indel detection methods.

Method	Reagents, consumables, and service fees per sample	Total money cost per sample	Time cost per sample
Barcode-pooled amplicon sequencing with IndelQuant (36-plex)	Barcoded sample preparation, $7–10; pooled library preparation, library QC, and sequencing modeled at $58 per pool, or $1.6 per sample	$8.6–11.6	Approximately 1 day for preparation; allow 1 weeks overall for planning, depending on sequencing availability
T7E1 assay	PCR, primers, cleanup, and electrophoresis consumables, $5–8; T7E1, $3.32	$14.82–21.32	Approximately 4–6 h; same-day completion
Sanger sequencing with TIDE/ICE	Sample preparation, $5–8; single-direction Sanger sequencing, $7.50	$16.50–23.00	Approximately 2–3 days, including preparation and submission
Cloning followed by Sanger sequencing (20 clones per sample)	Sample preparation, $5–8; cloning reaction, $17.45; culture and preparation of 20 sequencing templates, $25–45; plate-based sequencing, $52	$126.95–167.45	Approximately 3–5 days, including bacterial culture
Commercial amplicon sequencing service	sample preparation, $5–8; sequencing service at starting price of $58	$63–66	Approximately 1 day for preparation, followed by the advertised 8-business-day service turnaround

Supplementary Table S12. Runtime benchmarking and computing environment.

Dataset	RB	R1	R2
Barcode-defined entries submitted	30	36	36
Successfully completed entries	30	36	36
Merged reads entering CRISPResso2	797,978	533,221	620,626
Preprocessing time[1]	56 s	49 s	57 s
Total batch runtime	17 min 44 s	17 min 16 s	15 min 48 s
Mean CRISPResso2 runtime per successful entry	32.0 s	27.1 s	24.2 s
Batch runtime per successful entry[2]	35.5 s	28.8 s	26.3 s
Effective throughput[3]	102 entries/h	125 entries/h	137 entries/h

[1] Time from the first AdapterRemoval command to the first CRISPResso2 command, including demultiplexing, read merging, and intermediate-file handling.

[2] Total batch runtime divided by the number of successfully completed entries.

[3] Hardware configuration: Intel Core i5-12400F processor (6 physical cores, 12 logical processors), 16 GB RAM, and a 500 GB SSD containing the input and output files. The operating system is 64-bit Windows 10 Pro, version 22H2, build 19045.

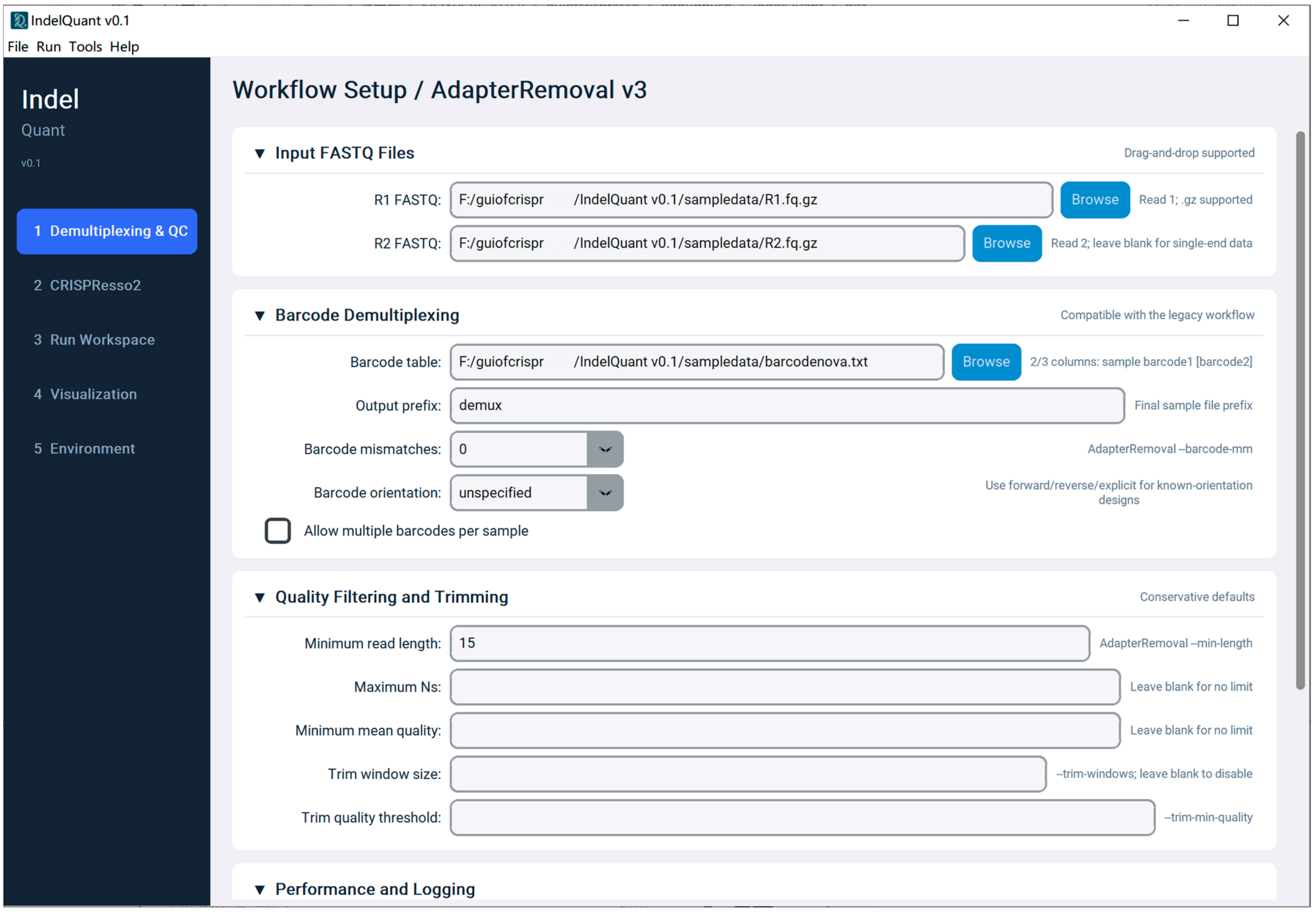

Supplementary Figure S1. Graphical user interface of IndelQuant – Demultiplexing tab.

The IndelQuant provides a graphical workflow setup panel for raw FASTQ input, barcode-based demultiplexing, quality filtering, read trimming, and paired-end read processing. Users can select R1 and R2 FASTQ files, import a barcode table, define barcode-mismatch and orientation parameters, and set basic quality-control thresholds without command-line operation. The left navigation panel organizes the full analysis into demultiplexing and quality control, CRISPResso2 configuration, run workspace, visualization, and environment management modules.

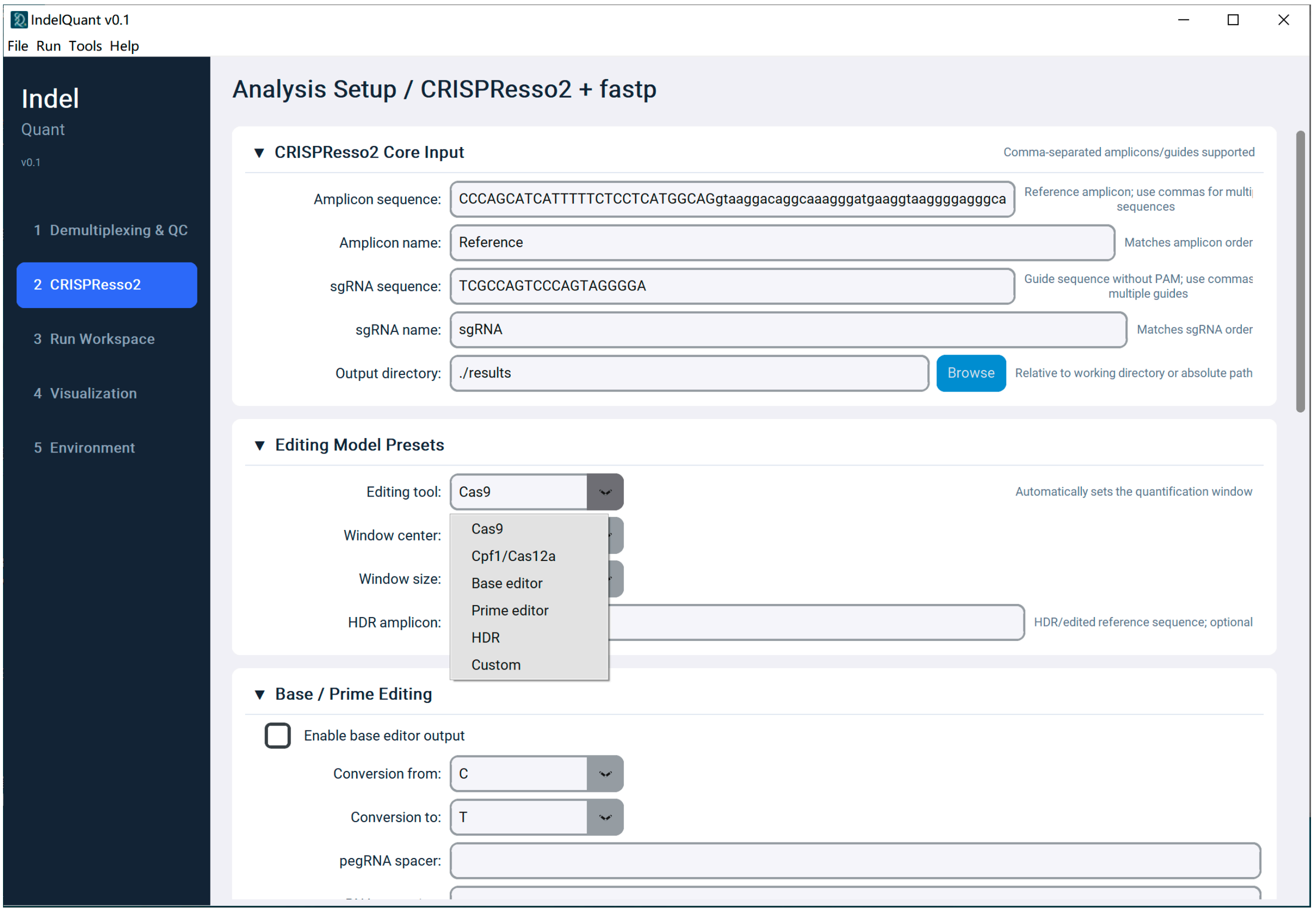

Supplementary Figure S2. Graphical user interface of IndelQuant – CRISPResso2 tab.

IndelQuant provides a graphical CRISPResso2 configuration panel for defining target sequences, selecting editing models, and setting analysis parameters. Users can enter the amplicon reference and sgRNA sequences, select presets for Cas9, Cpf1/Cas12a, base editing, prime editing, or HDR, and adjust alignment and quantification settings without command-line operation. Dedicated menus provide options for nucleotide conversions, pegRNA sequences, and nicking guides, along with process counts and output reports.

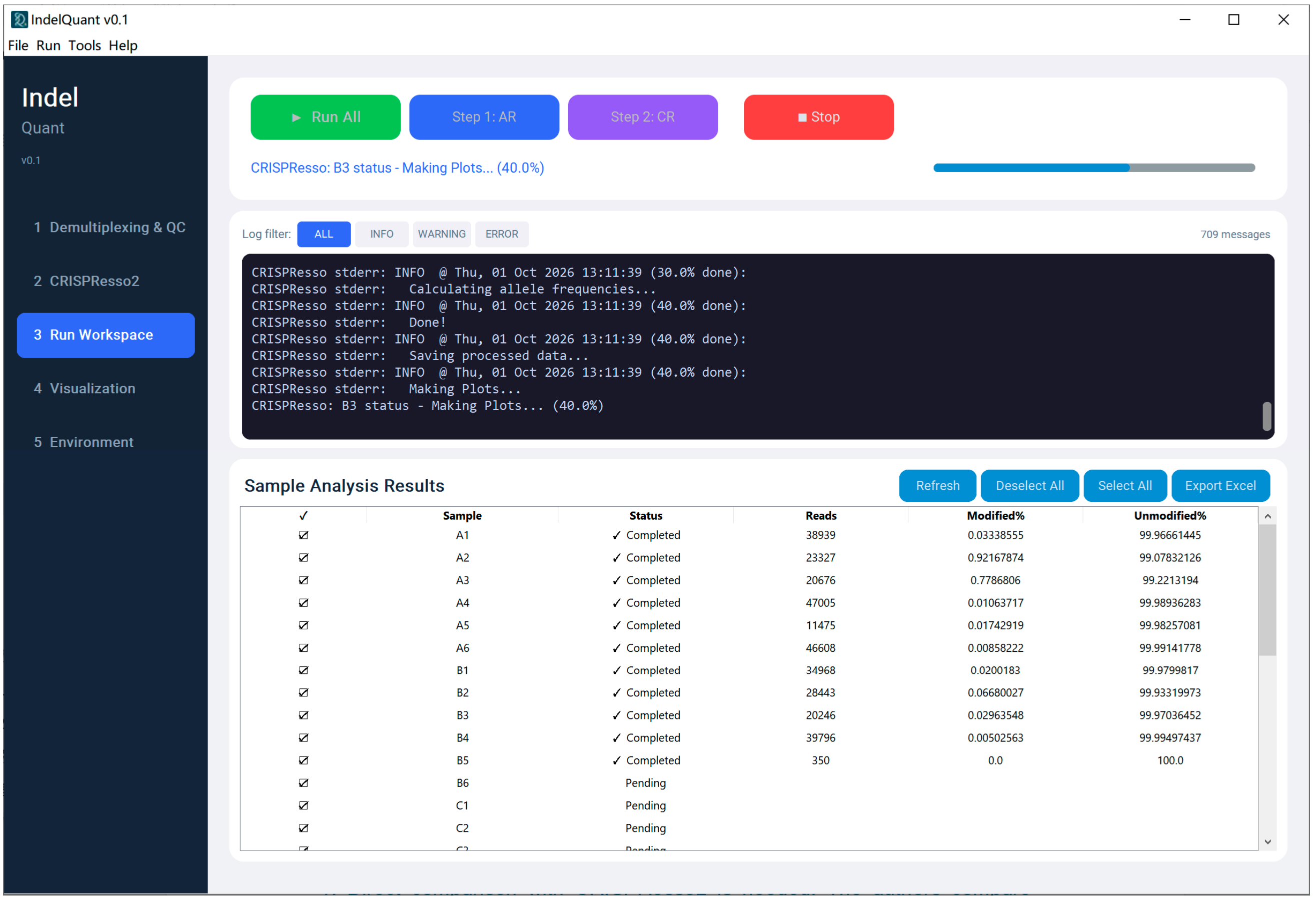

Supplementary Figure S3. Graphical user interface of IndelQuant – Run workspace.

IndelQuant provides a run workspace for managing analysis projects, individual samples, and their results. Users can select projects to run and inspect sample-level outputs within the interface. Progress bars and command-line logs support run monitoring and process management, while error messages help identify execution issues. Results are automatically exported as CSV files to the working folder and can also be exported separately by the user.

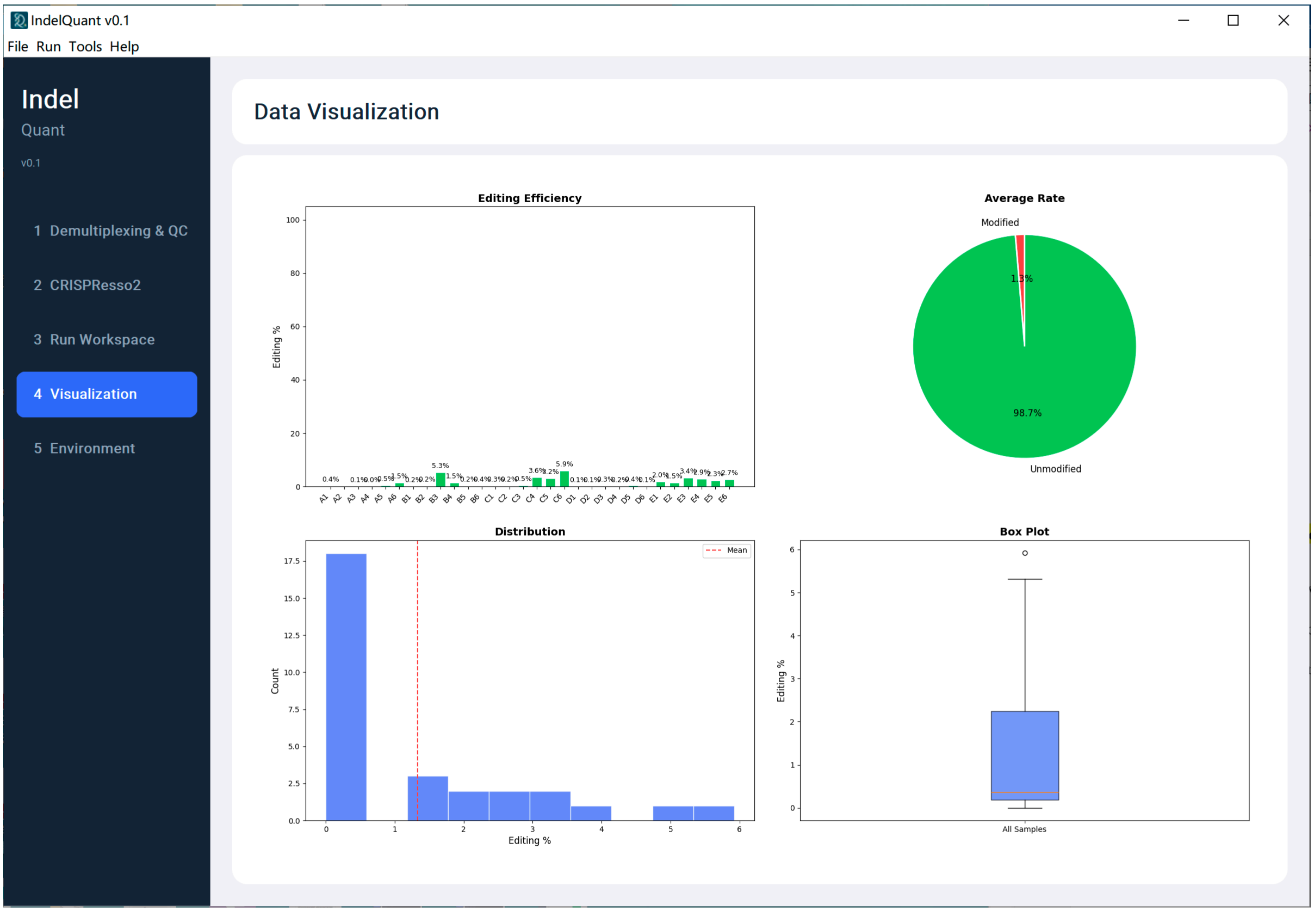

Supplementary Figure S4. Graphical user interface of IndelQuant - Visualization.

IndelQuant provides a visualization panel displaying bar plots, box plots, pie charts, and distributions of editing efficiencies across samples. These plots offer an overview of the dataset and facilitate comparisons among samples, particularly when analyzing large sample sets.

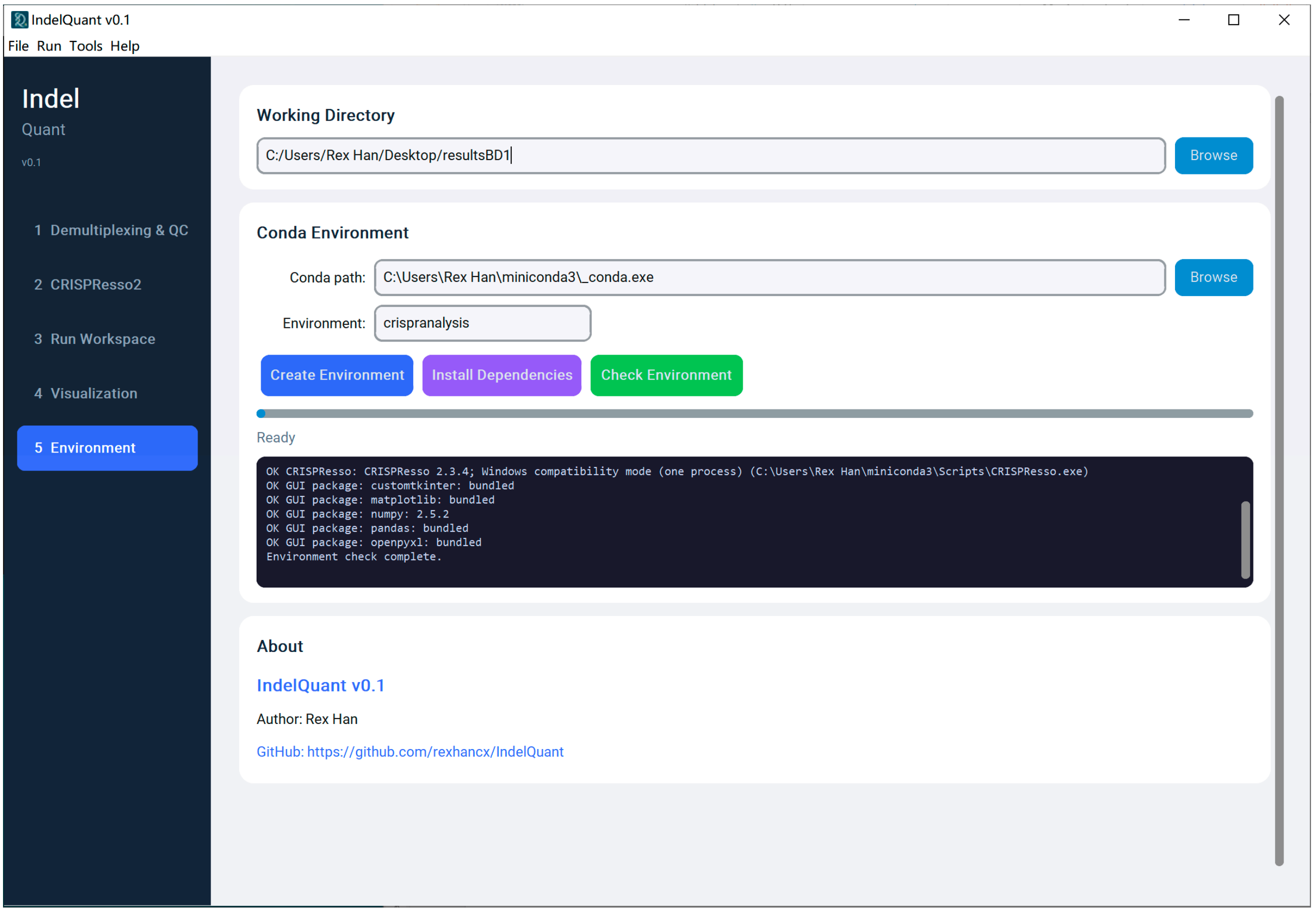

Supplementary Figure S5. Graphical user interface of IndelQuant - Environment.

The environment management tab in IndelQuant allows users to select the working directory, create the analysis environment, and install dependencies, CRISPResso2, and AdapterRemoval. Built-in checks verify the availability and configuration of the required components, helping identify setup issues before analysis.

pAV[CRISPR]-hCas9:P2A:EGFP-U6>gRNA3

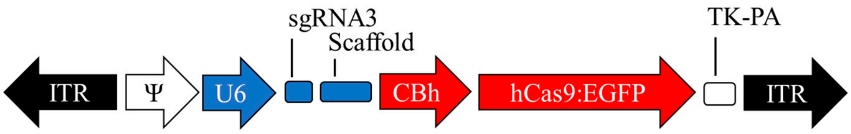

pAV[Exp]-CBh>hCas9(ns):P2A:EGFP

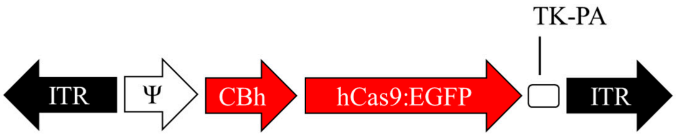

Supplementary Figure S6. Structures of recombinant adenoviral CRISPR-Cas9 vector constructs used for Ad5 production.

The TYR-targeting vector construct was packaged to generate Ad5-Cas9/gRNA3 viral particles, which deliver SpCas9, TYR-targeting gRNA3, and EGFP. The non-targeting control vector construct was packaged to generate Ad5-Cas9/ns viral particles, which deliver SpCas9 and EGFP without a TYR-targeting gRNA. The adenoviral particles generated from these constructs, rather than the plasmid constructs themselves, were used for postnatal jugular-vein delivery and embryonic dorsal-aorta delivery experiments.

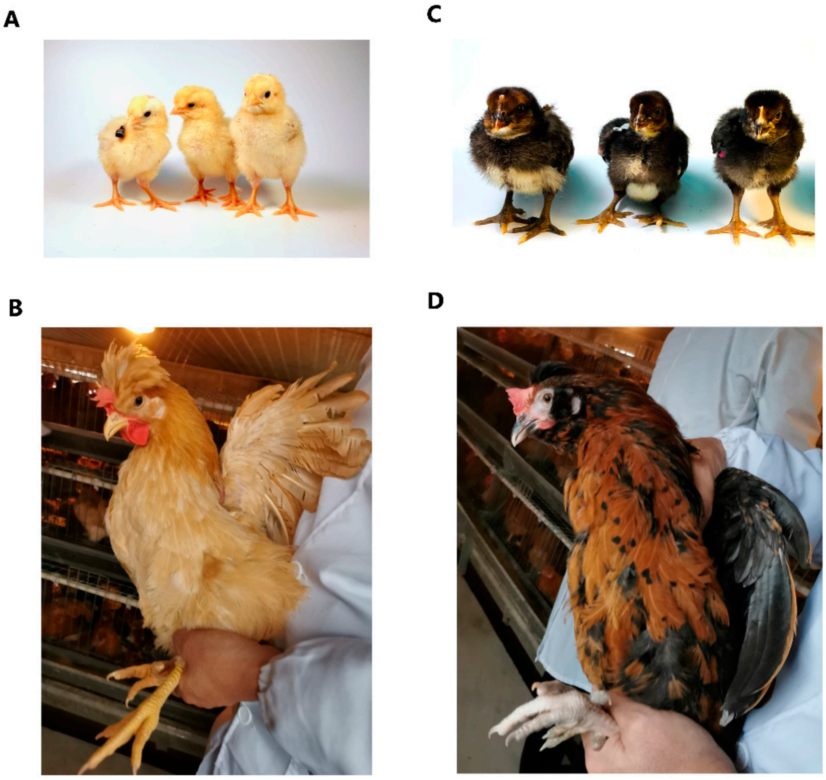

Supplementary Figure S7. ***TYR*-edited chimeric chickens generated by adenoviral CRISPR-Cas9 delivery.**

(A) Beijing You chicks. (B) Beijing You adult chickens. (C) Xinyang Black chicks. (D) Xinyang Black adult chickens.
